# Sweet syndrome following cervicofacial rhytidectomy: A case report

**DOI:** 10.1016/j.jpra.2026.07.023

**Published:** 2026-07-23

**Authors:** B. Vais, K. Serror, B. Pulvermacker, E. Zakine, J-D. Bouaziz, M. Chaouat, D. Boccara

**Affiliations:** a Plastic and reconstructive surgery Department, Hôpital de la Cavale Blanche, Brest, France; b Plastic and reconstructive surgery Department, Hôpital Saint-Louis, Paris, France; c Dermatology Department, Hôpital Saint-Louis, Paris, France

**Keywords:** Sweet syndrome, Facelift, Neutrophilic dermatoses, Aesthetic surgery, Plastic surgery, Corticosteroid therapy

## Abstract

Sweet syndrome is a rare inflammatory disorder characterized by fever, leukocytosis, and painful erythematous-violaceous plaques associated with a neutrophilic dermal infiltrate. This condition has never been described in the setting of aesthetic surgery.

We report the case of a 60-year-old woman who developed Sweet syndrome following a cervicofacial rhytidectomy combined with blepharoplasty and lower eyelid lipofilling. The favorable outcome under systemic corticosteroid therapy highlights the importance of early diagnosis in order to avoid unnecessary surgical procedures.

## Introduction

First described by Robert Sweet in 1964,^1^ Sweet syndrome, also known as acute febrile neutrophilic dermatosis, is a rare condition with an annual incidence of 3 cases per million patients. It may occur idiopathically, as a paraneoplastic manifestation, drug-induced, or more rarely, in the postoperative setting.

Several cases of Sweet syndrome occurring after surgery have been reported, notably following hand surgery,^2^ pneumonectomy,^3^ spinal surgery,^4^ or breast reconstruction using a DIEP flap.^5^ However, no case has been reported after Facelift or any other aesthetic surgical procedure.

The diagnosis of Sweet syndrome is established when at least two major criteria and two minor criteria are met^6^:

## Major criteria

1. Sudden onset of painful erythematous plaques or nodules.
2. Histologic evidence of a dense infiltrate of mature neutrophils in the dermis, without true leukocytoclastic vasculitis.

## Minor criteria

1. Fever above 38°C.
2. Association with a triggering factor: infection, inflammatory disease, neoplasia, pregnancy, drug exposure, or surgery.
3. Leukocytosis with predominant neutrophilia.
4. Rapid response to corticosteroids or anti-inflammatory therapy.

The differential diagnosis mainly includes postoperative bacterial infections, Pyoderma gangrenosum (PPG), and ischemic skin necrosis. However, the negative microbiological samples systematically obtained in this setting, together with the favorable response to corticosteroid therapy, strongly support the diagnosis of a neutrophilic dermatosis.

We report here the first documented case of Sweet syndrome following cervicofacial rhytidectomy, diagnosed early and successfully treated with corticosteroid therapy.

## Clinical background

A 60-year-old woman, whose only medical history was Hashimoto's thyroiditis, with no history of malignancy or immunosuppressive treatment, underwent cervicofacial rhytidectomy with SMAS plication combined with lower blepharoplasty and lower eyelid lipofilling.

**Fig. 1:**
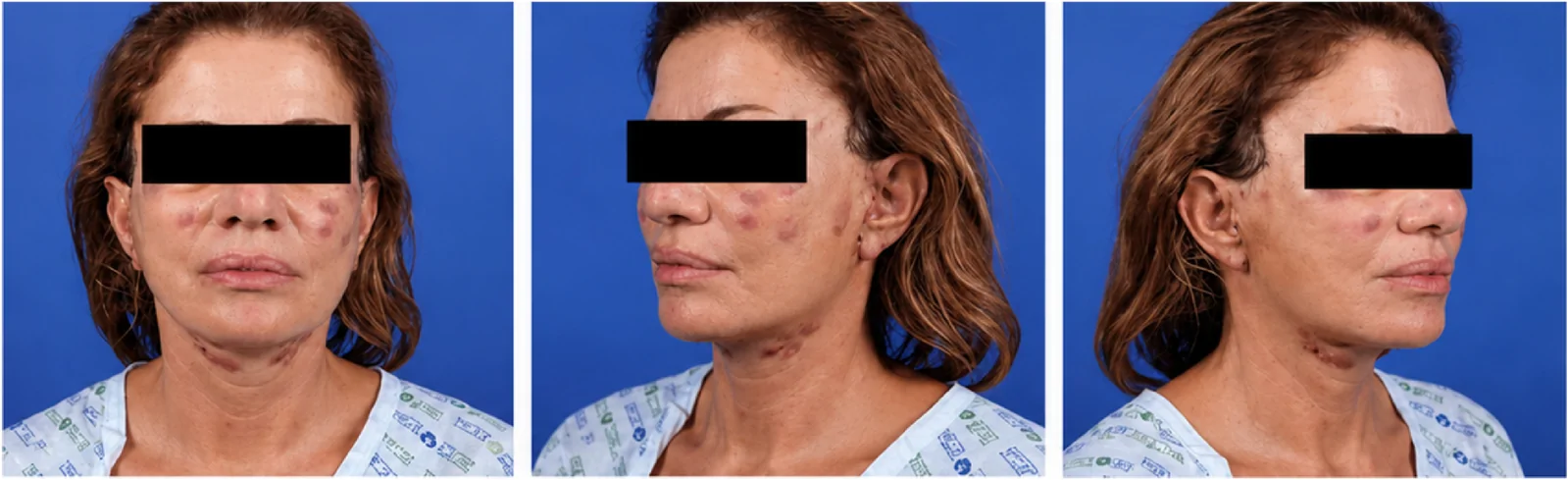
Clinical photographs (frontal and bilateral oblique views) showing characteristic erythematous infiltrated facial and cervical plaques of Sweet syndrome.

The procedure was uneventful, and the immediate postoperative course was satisfactory until postoperative day 2, when the patient developed painful malar and cervical erythema (Fig. 1), associated with moderate fever (38°C). Her primary surgeon prescribed empiric antibiotic therapy with Amoxicillin/clavulanic acid for suspected infectious cellulitis.

The absence of improvement and the rapid progression of the lesions into edematous erythematous-violaceous plaques led to her hospitalization on postoperative day 6 in our referral center. On admission, clinical examination revealed infiltrated erythematous plaques, painful on palpation, involving the malar, cervical, dorsal, and upper chest regions, without fluctuation or purulent discharge. There was no skin necrosis or wound dehiscence.

Body temperature was 38°C, with a white blood cell count of 9.03 G/L and elevated C Reactive protein at 75 mg/L. Bacteriological, virological, blood, and skin samples were obtained and all returned negative. Histologic examination of a skin biopsy showed a dense dermal infiltrate of neutrophils without leukocytoclastic vasculitis, confirming the diagnosis of Sweet syndrome.

**Fig. 2:**
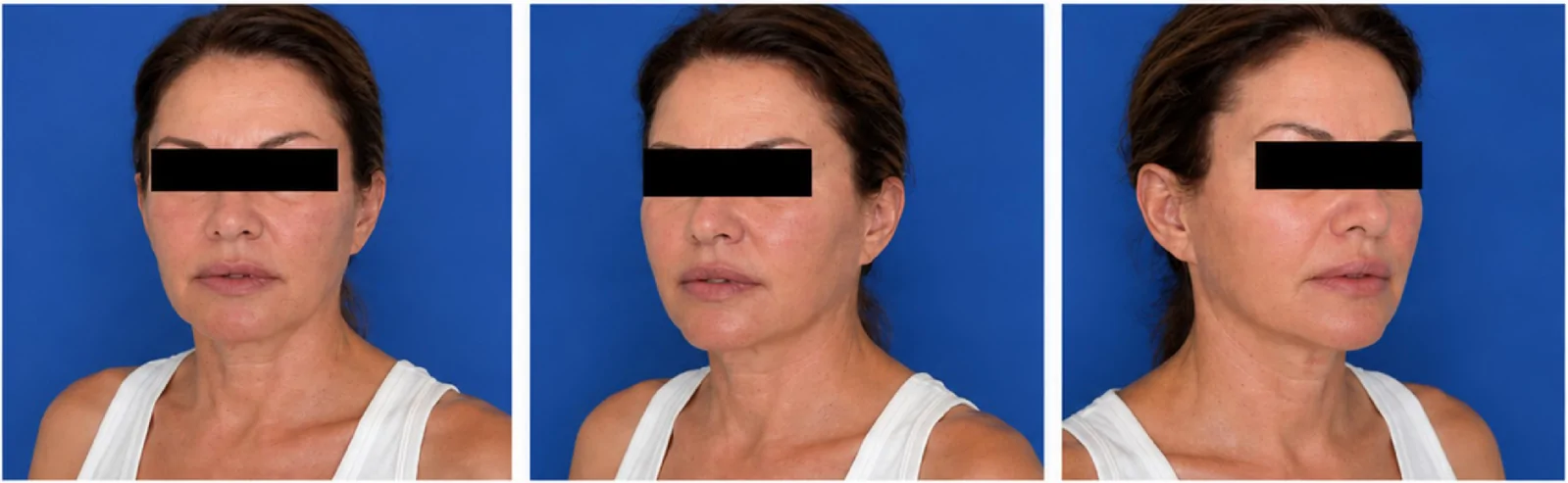
Clinical photographs 3 months after onset of Sweet syndrome showing marked resolution of facial and cervical lesions.

Systemic oral corticosteroid therapy was initiated at a dose of 1 mg/kg/day, resulting in clinical improvement within 48 hours, with resolution of fever and progressive regression of the plaques. No additional surgical intervention was required. The scars remained supple, without dehiscence or signs of infection. At three-month follow-up, the patient showed complete healing, a satisfactory aesthetic outcome, and no recurrence (Fig. 2).

## Discussion

Sweet syndrome was initially described as an acute inflammatory disorder characterized by painful cutaneous lesions associated with fever and a dense neutrophilic dermal infiltrate.^1^ Although it is classically considered an acute condition that responds favorably to systemic corticosteroid therapy, several studies have shown that its course may be associated with cutaneous and systemic sequelae, particularly in severe or untreated forms.

From a cutaneous perspective, post-inflammatory dyschromia, atrophic or hypertrophic scarring, and ulceronecrotic lesions have been reported, especially in bullous or necrotic variants associated with intense neutrophilic infiltration and sometimes secondary vasculitis.^6^^,^^7^ Most patients heal without scarring; however, chronic or recurrent forms may occur.^6^ Cohen and Kurzrock also described recurrences in a significant proportion of cases, which may lead to persistent cutaneous morbidity and impaired quality of life.^7^^,^^8^

Extracutaneous involvement represents another potential source of sequelae. Ocular, pulmonary, neurologic, and articular manifestations have been described and may compromise functional prognosis in the absence of prompt treatment.^7^^,^^9^ These systemic manifestations are likely related to widespread neutrophil activation and increased production of pro-inflammatory cytokines such as Interleukin-1 beta, Interleukin-6, and Granulocyte colony-stimulating factor, which are thought to play a central role in the pathophysiology of the disease.^9^

Furthermore, association with an underlying disorder, particularly hematologic disease or malignancy, constitutes a major indirect prognostic concern and justifies prolonged surveillance. Hematologic malignancies, especially Acute myeloid leukemia and

Myelodysplastic syndromes, are the most frequently reported associations, supporting the role of Sweet syndrome as a potential paraneoplastic syndrome.

In the context of plastic surgery, Sweet syndrome represents a major diagnostic challenge.

Particular attention should be paid to the differential diagnosis with postoperative PPG, another rare neutrophilic dermatosis that may occur after surgical procedures and closely mimic surgical site infection. While Sweet syndrome typically presents with painful erythematous plaques, fever, and a dense neutrophilic dermal infiltrate without vasculitis, PPG is characterized by rapidly progressive ulcerative lesions with violaceous undermined borders and tissue necrosis. Distinguishing between these conditions is of major clinical importance because surgical debridement, often performed when infection is suspected, may trigger pathergy and dramatic lesion worsening in patients with PPG. Conversely, Sweet syndrome generally presents with non-ulcerative inflammatory plaques and responds rapidly to systemic corticosteroids. In both conditions, negative microbiological cultures, lack of response to antibiotics, and early dermatologic assessment should prompt consideration of a neutrophilic dermatosis. Awareness of these entities is crucial for plastic surgeons, as delayed diagnosis may result in unnecessary surgical procedures, prolonged hospitalization, impaired aesthetic outcomes, and increased morbidity.

Several postoperative cases have been reported following thoracic, spinal, or breast reconstructive surgery, highlighting the diagnostic difficulty in the surgical setting and the importance of early recognition.

These observations support the systematic inclusion of Sweet syndrome in the differential diagnosis of postoperative cutaneous complications, particularly in cases of atypical progression or failure of well-conducted antibiotic therapy. Early skin biopsy and prompt initiation of systemic corticosteroid therapy are key elements of management in order to limit cutaneous and functional sequelae.

The current literature is mainly based on observational studies, case series, and case reports, limiting the level of evidence regarding the true frequency of sequelae and prognostic factors. Few prospective or randomized studies are available, and the pathophysiology remains only partially understood. Future studies may help identify predictive biomarkers of severity and recurrence, as well as assess the comparative efficacy of emerging immunomodulatory therapies, particularly IL-1 inhibitors.

Thus, Sweet syndrome represents a rare but potentially severe dermatologic complication in plastic surgery, whose early recognition is essential to prevent cutaneous and systemic sequelae and optimize therapeutic management.

## Conclusion

Sweet syndrome following cervicofacial rhytidectomy is a postoperative diagnosis of exclusion whose frequency is exceptional but important to recognize. Any painful, febrile erythematous eruption unresponsive to antibiotics should raise suspicion for this condition. Skin biopsy confirms the diagnosis, and corticosteroid therapy generally leads to rapid resolution. Early diagnosis is essential to avoid unnecessary reoperations and preserve aesthetic outcomes.

## Ethical approval

Not required.

## Funding

None.

## Declaration of competing interest

None declared.
